# Case Report: Diagnosis and management of primary ovarian squamous-cell carcinoma: a report of two cases and systematic review of the literature

**DOI:** 10.3389/fonc.2026.1706736

**Published:** 2026-01-23

**Authors:** Feifei Guo, Bingna Huang, Yue Hua, Kang Zheng, Jing Chen, Ling Chen, Xiujuan Jing, Rong Li, Huaijun Zhou

**Affiliations:** 1 Department of Gynecology, Nanjing Drum Tower Hospital, Affiliated Hospital of Medical School, Nanjing University, Nanjing, China; 2 Clinical College of Nanjing University of Chinese Medicine, Nanjing Drum Tower Hospital, Nanjing, China; 3 The Affiliated Hospital of China Pharmaceutical University, Pukou Traditional Chinese Medicine Hospital, Nanjing, China

**Keywords:** complete cytoreduction, ovarian cancer, pathology, primary ovarian squamous-cell carcinoma, prognosis

## Abstract

**Objective:**

To present two cases of primary ovarian squamous-cell carcinoma (POSCC) and describe a comprehensive review and analysis of the published related literature.

**Methods:**

We reviewed the medical records of two patients with POSCC who underwent optimal debulking surgery followed by systemic chemotherapy at Nanjing Drum Tower Hospital. We also reviewed published case reports and case series describing POSCC, focusing on histogenesis, diagnostic approaches, current therapeutic modalities, and prognosis associated with this condition.

**Results:**

The median age at diagnosis of patients with POSCC was 53.4 years. Few patients were diagnosed at an early stage (stage I, 19.71%), and tumor stage had a significant prognostic effect (*p* = 0.0081). However, the difference in survival between advanced stages (stage III and stage IV) was small and not statistically significant. Most patients underwent hysterectomy and bilateral salpingo-oophorectomy, and omentectomy and lymphadenectomy improved survival outcomes in patients with advanced-stage disease.

**Conclusion:**

Primary ovarian squamous-cell carcinoma is a rare and challenging type of ovarian cancer, and its prognosis remains extremely poor. Aggressive multimodal treatment may include surgery, systemic chemotherapy, and targeted therapies.

## 1 Introduction

Primary ovarian squamous-cell carcinoma (POSCC) is an exceptionally rare and aggressive malignancy, accounting for less than 1% of all ovarian cancers. Current understanding of this entity remains limited because of its low incidence and the scarcity of robust clinical evidence, with existing literature predominantly relying on small case series (cumulative n < 100) and isolated reports. This knowledge gap underscores the need for multinational registry studies to establish consensus on disease behavior and prognostic determinants.

To elucidate clinicopathological patterns and therapeutic correlations, we conducted a dual analytical approach: (i) a retrospective evaluation of two POSCC cases managed at Nanjing Drum Tower Hospital, and (ii) a systematic synthesis of globally published evidence on POSCC.

## 2 Materials and methods

Written informed consent was obtained from both patients, and the present study was approved by the Institutional Review Board of Nanjing Drum Tower Hospital.

A systematic literature review was conducted using PubMed, Google Scholar, and the China National Knowledge Infrastructure (CNKI), covering publications from January 1964 to December 2022. Boolean operators (*AND/OR*) were used to combine the following search terms: “*primary squamous cell carcinoma of ovary*,” “*ovarian neoplasms*,” and “*SCC of ovary*.” No language restrictions were initially imposed.

For eligible cases, we extracted standardized variables, including demographic and clinicopathological characteristics; tumor laterality (unilateral or bilateral) and anatomical localization; preoperative diagnostic modalities (imaging and biomarker profiles); surgical approaches and adjuvant treatment regimens; and survival outcomes with follow-up duration. Publications in languages other than English or Chinese were subsequently excluded to reduce the risk of translational inaccuracies during data synthesis.

## 3 Statistical analysis

Data management and statistical analysis were conducted using GraphPad Prism 9. Survival curves were generated to estimate overall survival (OS), with survival duration calculated from the date of primary surgical intervention. Differences in survival rates were assessed using the log-rank test. Pearson’s product–moment correlation coefficient (r) was calculated to quantify linear relationships. A p-value <0.05 was considered statistically significant for all inferential analyses.

## 4 Case presentation

### 4.1 Case 1

A 65-year-old postmenopausal multiparous woman (G3P1) presented to our gynecology clinic in December 2020 with a pelvic mass identified during routine health screening. Transvaginal ultrasonography revealed a heterogeneously echogenic left adnexal mass measuring 86×64×52 mm, demonstrating mixed solid–cystic architecture with irregular septations. Preoperative tumor marker profiling showed normal CA125 (20 U/mL; reference <30.2 U/mL) and squamous cell carcinoma (SCC) antigen levels (1.95 ng/mL; reference <2.5 ng/mL). The patient’s medical history was significant for essential hypertension and atherosclerotic coronary artery disease.

**Figure 1:**
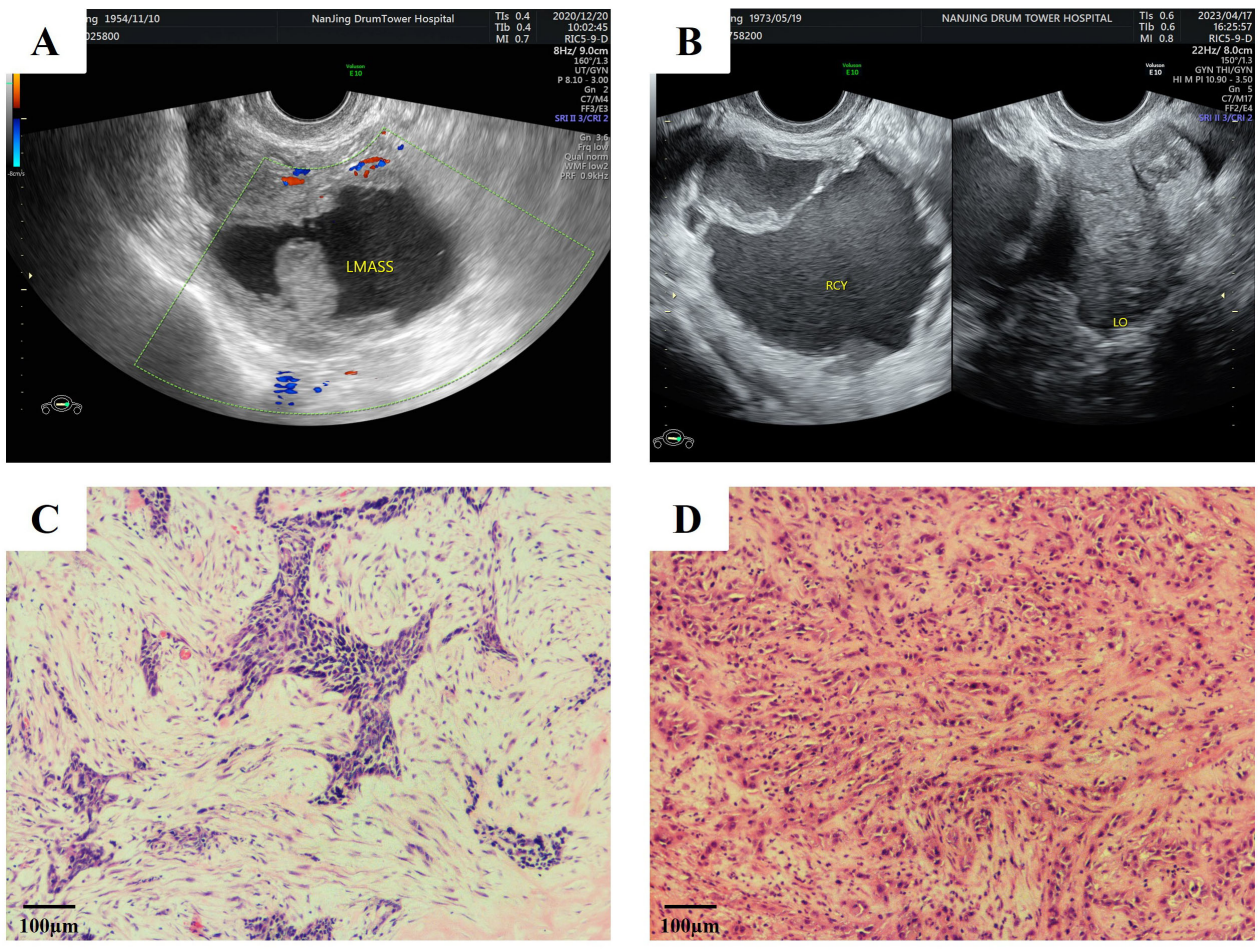
The ultrasound findings and pathological diagnosis results of two patients. **(A, B)** Routine gynecological ultrasound examination: **(A)** A mixed echogenic lesion measuring 86×64×52 mm is identified in the left pelvic region, predominantly cystic with dense internal echogenic foci, enclosed by an intact capsule measuring 2.4 mm in thickness (case 1). **(B)** A cystic anechoic area measuring 81×69×62 mm is observed in the right ovary, containing dense echogenic foci and septations, enclosed by an intact capsule measuring 1.3 mm in thickness. The left ovary measures 19×22×11 mm (case 2). **(C, D)** Tumor cells were pleomorphic, and mitotic figures were numerous. (H&E, x100). **(C)** Ovarian squamous cell carcinoma, moderately to poorly differentiated (case 1). **(D)**. Ovarian malignant epithelial tumor composed of squamous cell carcinoma (approximately 90%), mucinous adenocarcinoma (approximately 5%), and clear cell carcinoma (approximately 5%), consistent with ovarian mixed carcinoma, moderately to poorly differentiated. Note: Extensive sampling revealed no definitive evidence of teratomatous components, endometriotic components, or benign/borderline Brenner tumor components.

**Figure 2:**
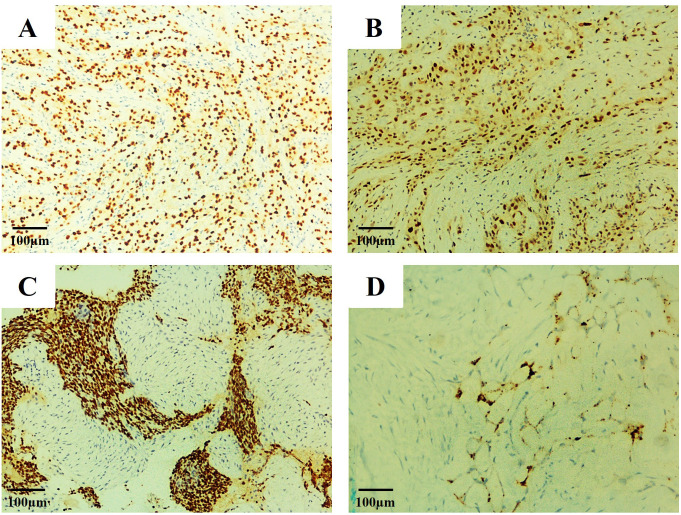
Immunohistochemical staining of tumor tissue. **(A)** P40, **(B)** P53, **(C)** P63 and **(D)** NapSinA. (H&E, x100).

Surgical intervention comprised total hysterectomy, bilateral salpingo-oophorectomy, omentectomy, and rectectomy. Pathological examination showed that the tumor in the left ovary consisted of solid and cystic components. The cut surface of the left ovary was grayish white and predominantly solid, with papillary structures attached to the inner wall of the cyst. The left fallopian tube was unremarkable. The uterus and right adnexa, as well as the peritoneum and omentum, were intact and free of metastasis. Suspected partial involvement of the rectal serosal surface by tumor was observed. Histopathological evaluation confirmed poorly differentiated POSCC of the left ovary, with invasion of the rectal serosal surface. Surgical margins from the cervix, endometrium, and rectum were negative for malignancy, and no metastases were detected in other excised specimens. Final staging was FIGO stage IIIC (**Figures 1**, **2**).

Postoperative management included eight cycles of platinum-based adjuvant chemotherapy (carboplatin plus paclitaxel). Surveillance imaging at 14 months of follow-up detected a 4.3 cm right renal mass; nephron-sparing resection with concurrent retroperitoneal tumor debulking was subsequently performed. Histopathology confirmed metastatic squamous cell carcinoma, prompting second-line therapy with dose-dense paclitaxel plus bevacizumab for six cycles.

Nearly 4 years after diagnosis, disease progression was detected by PET/CT in October 2024, demonstrating new hepatic and diaphragmatic metastases (largest lesion, 4.6×4.5 cm). The patient underwent right radical nephrectomy with wedge hepatectomy and diaphragmatic plaque resection, followed by third-line chemotherapy with carboplatin, paclitaxel, and bevacizumab. Histopathological review of resected specimens confirmed poorly differentiated SCC metastases. The patient elected to continue maintenance bevacizumab with ongoing disease surveillance.

### 4.2 Case 2

A 50-year-old postmenopausal multiparous woman (G3P1) presented to our gynecologic oncology unit in April 2023 with progressive enlargement of a right pelvic mass. Transvaginal sonography demonstrated a complex right adnexal lesion measuring 81 × 69 × 62 mm, exhibiting heterogeneous echotexture with multiple septations and solid components. Serum tumor marker analysis revealed elevated CA125 (142.3 U/mL; reference <30.2 U/mL) and CA19-9 (39.9 U/mL; reference <27 U/mL), while SCC antigen levels remained within normal limits (1.59 ng/mL; reference <2.5 ng/mL). The patient’s medical history was notable for chronic hepatitis B virus infection and high-risk human papillomavirus (HPV 58) carriage, without other comorbidities.

Initial surgical management involved diagnostic laparoscopy with right salpingo-oophorectomy. Pathological examination showed that the tumor in the right ovary consisted of cystic components; the cut surface was grayish white with hemorrhagic areas and patchy necrosis. A right salpingitis abscess was also identified. Histopathological analysis revealed a mixed epithelial ovarian malignancy comprising three distinct components: poorly differentiated primary ovarian squamous cell carcinoma (POSCC, 90%), mucinous adenocarcinoma (5%), and clear cell carcinoma (5%) (**Figures 1**, **2**).

Because of family-related reasons, adjuvant chemotherapy and further surgery were delayed. Four weeks later, a staging laparotomy (including total hysterectomy with left salpingo-oophorectomy, infragastric omentectomy, and en bloc resection of pelvic tumor deposits) was performed. Intraoperative exploration revealed extensive carcinomatosis involving the pelvic peritoneum, rectosigmoid muscular layer, and right parametrium, with associated pyogenic abscess formation. Final histopathology confirmed FIGO stage IV, grade 2/3 POSCC with transmural uterine involvement, omental metastases, and full-thickness rectal wall infiltration. Notably, no teratomatous elements, Brenner tumor components, or endometriotic foci were identified.

Postoperative adjuvant therapy consisted of dose-dense weekly paclitaxel with carboplatin for five cycles. Treatment was discontinued because of grade 3 hematologic toxicity (neutropenic fever and platelet count <50 × 10^9^/L) and progressive disease evidenced by new hepatic metastases. The patient elected to pursue palliative care following informed consent discussions and ultimately died from disease progression 9 months after the initial diagnosis.

## 5 Results

### 5.1 Characteristics of participants

Our systematic review included 75 histologically confirmed cases of POSCC that met predefined inclusion criteria, comprising (i) two novel cases from our center and (ii) 73 cases extracted from 42 eligible studies published between 1964 and 2022, with individual-level data available for at least 80% of critical variables. The evidence base consisted predominantly of single-case reports (n = 36; 85.7%), small case series (n = 6), and dedicated histomorphological analyses (n = 1).

A total of 310 publications were excluded, primarily because of metastatic SCC, incomplete documentation of critical variables or non-quantifiable outcome reporting (**Supplementary Figure 3**) (**Table 1**).

**Table 1:** Cases of pure primary squamous cell carcinoma of ovary reported in the literature.

Cases	References	Age (years)	Site	FIGO stage/grade	Surgery	Adjuvant therapy	Outcome/follow-up (months)
1	Black Benitz	35	U	I/1	H, BSO	No	NR
2	Shingleton	54	U	I/1	RO	RT	Died, 6
3	Macko	90	U	I/2	UO	No	Alive, 30
4	Chen	49	U	I/1	H, BSO	RT	Alive, 12
5	Ben-Brauch	65	U	III/2	H, BSO, ileectomy, TD	CT	Died, 6
6	Yetman	33	U	I/2	H, BSO, PLND, PALND, AP	No	Alive, 15.6
7	Kashimura	61	U	II/NR	H, BSO	CT, RT	Died, 9
8		42	U	III/NR	LSO	RT	Died, 8
9		50	U	I/NR	H, BSO	RT	Alive, 14 years
10	Radhi	64	B	IV/2	TD	No	Died, 9 days
11	Mc Grady	53	B	I/1	H, BSO	No	Alive,12
12	Pins	73	U	IIA/3	H, BSO	RT	Died, 49
13		61	U	IIB/3	H, BSO	CT, RT	Alive, 60
14		55	U	IIB/3	H, BSO, TD	CT	Alive, 30
15		36	U	IIC/3	H, BSO	CT	Died, 8
16		64	B	IB	RSO, LO	NR	Alive, 60
17		55	U	IIIB/3	H, BSO	CT	Died, 2
18		52	U	IIIC/3	Ovarian, omental biopsy	NR	NR
19		46	B	IIIC/3	Ovarian, omental biopsy	NR	NR
20		27	U	IIIC/3	H, BSO	CT	Died, 1
21		70	U	IIIC/3	H, BSO	CT	Died, 5
22		73	U	IV/3	LSO	RT	Died, 1
23	Mai	40	B	I/2	H, BSO	NR	NR
24	Khanfar	14	U	IV/3	BSO	CT	Died, 6
25	Balat	40	B	IB/NR	H, BSO, PLND, AP, O, right nephrectomy	CT	Died, 24
26	Chien	63	U	IV/3	H, BSO, PLND, O, TD	CT	Died, 7
27	Todo	56	U	IIIC/3	H, BSO, PLND, sigmoidectomy	CT	Died, 12
28	Amjad	31	U	IIIC/1	H, BSO, O, BR	CT	Alive, 3
29	Park	76	U	IIC/1	H, BSO, PLND, O, AP	CT	Alive, 90
30		48	NR	IV/2	H, BSO, PLND, O, AP	CT	Alive, 9
31	Shakuntala	50	U	IIC/2	H, BSO, PLND	CT, RT	Died, 6
32	Nandedkar	28	U	IIB/2	H, LSO	CT	Died, 2
33	Vidyadhara	45	B	NR/2	BO	NR	NR
34	Park	46	U	IVB/2	H, BSO, TD, BR	CT	Died,12
35	Sworn	39	U	I/3	H, BSO	No	Alive, 60
36	Genandry	41	B	I	H, BSO	No	NR
37	Sonam Sharma	66	U	IIIC/2	H, BSO, O, right parietal wall mass removal	CT, RT	Died, 2
38	Nakamura	71	U	IIB/NR	RSO, LO	CT	Alive, 18
39	Shrivastava	30	U	IIIC/NR	H, BSO, O, ileectomy	CT	Died, 12
40	Mimura	50	U	IIB/3	H, BSO, PLND, PALND, O, BR	CT	Alive, 7
41	Koufopoulos	55	U	IIIC/3	H, BSO	CT	Died, 9
42	Zain abid	35	U	IVB/2	H, BSO, AP	CT	NR
43	Tang	45	U	IIIC/2	H, BSO, PLND, PALND, O	CT	Alive, 42
44		43	U	IIB/3	H, BSO, PLND, PALND, O	CT	Alive, 9
45	Jie	63	U	IIB/3	H, BSO, O, colectomy	CT	Died, 3
46	Kong	56	U	NR	H, BSO, O, ileectomy	CT, Cryo-assisted operation	Alive, 14
47	Eltabbakh	31	U	IV/3	H, BSO, O, colectomy	CT	Alive, 24
48	Yan Luo	52	U	IIIA/3	H, BSO, PLND, PALND, O, AP, multiple peritoneal biopsies	CT	Died, 11
49	Hiroyuki	71	NR	IIIC	LO, RSO, BR	CT	Alive,60
50	Sharma	66	U	IIIC/2	BSO, O,	CT, RT	Died, 2
51	Yukiharu	56	NR	IIIC	H, BSO	CT	Died, 11
52	Tobar	28	U	NR	TD	CT	Died, 15
53		31	NR	IV	TD	CT	Alive,12
54		56	NR	IIIC	TD	CT	Died, 11
55		31	NR	IIIC	H, BSO	CT	Died, 3
56	Yun Xi	61	B	IIIC/3	NR	NR	Died,42
57		70	U	IIB/2	NR	NR	Died,61
58		82	U	IA/3	NR	NR	Died,60
59		53	U	IA/3	NR	NR	Died,40
60		52	NR	IIIC/3	NR	NR	Died,71
61		84	NR	IIB/3	NR	NR	Died,4
62		64	U	IIB/2	NR	NR	Died,43
63		70	U	IVB/3	NR	NR	Died,9
64		74	U	IIIB/3	NR	NR	Died,40
65		46	U	IIIA2/3	NR	NR	Died,40
66		56	NR	IIIC/3	NR	NR	Died,15
67		67	U	IIIC/3	NR	NR	Died,2
68		59	NR	IIB/2	NR	NR	Died,12
69		54	NR	IIB/3	NR	NR	NR
70		61	NR	IIB/1	NR	NR	Died,12
71	Xu	63	U	IIIC/3	H, RSO, O, PLND, PALND, AP, BR	CT	Alive, 18
72	Qiao	50	U	III/NR	H, BSO	CT	Died, 6
73	Wang	63	U	IIB	H, BSO, O, BR	CT	Died, 3
74	Present 1	65	U	IIIC/3	TD	CT	Alive to now
75	Present 2	50	U	IV2/3	TD	CT	Died, 12

FIGO, International Federation of Gynecology and Obstetrics; H, Hysterectomy; BSO, Bilateral salpingo-oophorectomy; RO, Right oophorectomy; RT, Radiation therapy; CT, Chemotherapy; UO, Unilateral oophorectomy; BO, Bilateral oophorectomy; PLND, Pelvic lymph node dissection; LSO, Left salpingo-oophorectomy; TD, Tumor-cell debulking; RSO, Right salpingo-oophorectomy; LO, Left oophorectomy; PALND, Para-aortic lymph node dissection; AP, Appendectomy; NR, Not recorded; U, Unilateral; B, Bilateral; BR, Bowel resection; O, Omentectomy.

### 5.2 Age, tumor size, and stage

The cohort exhibited a non-normal age distribution (Shapiro–Wilk *p* < 0.01), with a mean age of 53.4 ± 15.0 years (range, 14–90 years) and a peak incidence in the 61–70-year age group (**Supplementary Figure 1**; **Supplementary Table 1**). Preoperative clinical manifestations predominantly included abdominal pain and palpable pelvic masses, with 23.6% of patients presenting with both symptoms.

**Figure 3:**
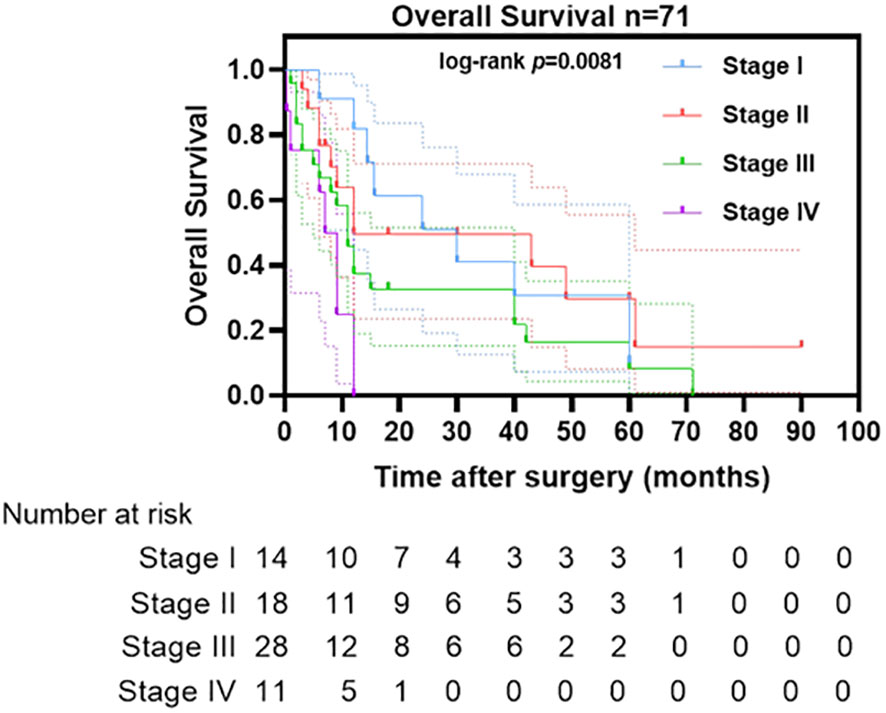
Overall Survival of patients grouped by tumor stage.

According to the revised 2014 FIGO staging system, early-stage disease (stage I) accounted for 19.44% of cases (14/72 evaluable). Advanced-stage disease (stages III–IV) comprised 54.17% of cases and demonstrated significant prognostic stratification (log-rank *p* = 0.0081; hazard ratio [HR] = 2.31). However, pairwise comparison between stage III (n = 28) and stage IV (n = 11) cohorts revealed no significant difference in survival (HR = 1.12, 95% CI 0.79–1.58; *p* = 0.51) (**Figure 3**). Multivariable analysis showed no clinically meaningful correlation between patient age and maximum tumor dimension (Pearson’s *r* = 0.1129, *p* = 0.43) (**Supplementary Figure 2**).

### 5.3 Surgical procedures and adjuvant therapy

Surgical management data stratified by tumor stage were available for analysis in 58 cases (**Table 2**). Primary cytoreductive procedures included radical hysterectomy with bilateral salpingo-oophorectomy (BSO) in 65.0% (38/58) of cases, therapeutic omentectomy in 32.8% (19/58), and systematic lymphadenectomy (pelvic and/or para-aortic) in 26.4% (15/58).

**Table 2:** Surgical treatment of patients with POSCC in relation to the FIGO stage.

Stage	Hysterectomy n (%)	Salpingo-oophorectomy n (%)			Omentectomy n (%)	Lymphadenectomy n (%)	Additional Surgery n (%)
		Bilateral	Unilateral	NR			
I	9/14 (64.29)	9/14 (64.29)	1/14 (7.14)	2/14 (14.29)	1/14 (7.14)	2/14 (14.29)	2/14 (14.29)
II	11/19 (72.58)	10/19 (52.63)	2/19 (10.53)	6/19 (31.58)	5/19 (26.32)	5/19 (26.32)	3/19 (15.79)
III	17/28 (60.71)	18/28 (64.29)	2/28 (7.14)	6/28 (24.42)	9/28 (32.14)	7/28 (25.00)	9/28 (32.14)
IV	7/11 (63.64)	8/11 (72.73)	1/11 (9.09)	1/11 (9.09)	6/11 (54.55)	4/11 (36.36)	4/11 (36.36)
Total	44/72 (61.11)	45/72(62.50)	6/72 (8.33)	15/72 (20.83)	21/72 (29.17)	18/72 (25.00)	18/72 (25.00)

Additional surgery comprises surgery to the bowel (14), and others (3). There was no record for 3 patients about FIGO stage.

**Figure 4:**
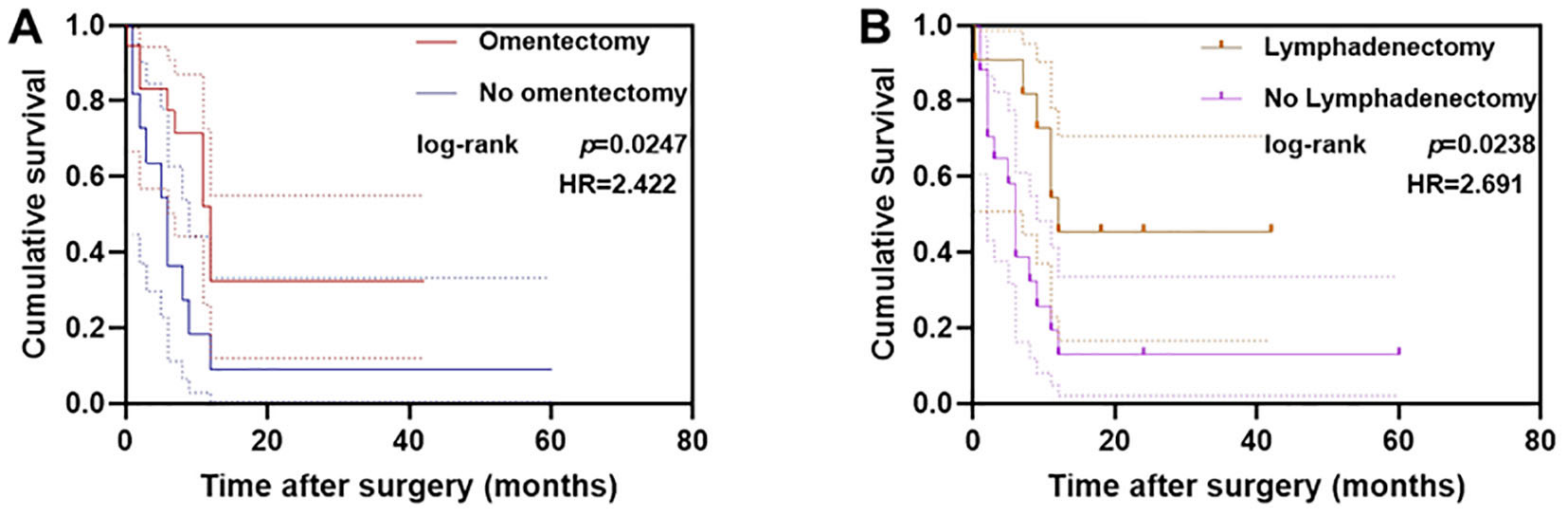
Survival in patients grouped by treatment with omentectomy **(A)** and lymphadenectomy **(B)**. The Kaplan-Meier curves show the overall survival probability over time, with log-rank test p-values indicating significant differences between groups.

Multivariable Cox regression analysis demonstrated improved overall survival (OS) among patients with advanced-stage disease who underwent omentectomy (hazard ratio [HR] = 2.422, *p* = 0.0247) and lymphadenectomy (HR = 2.691, *p* = 0.0238). This survival benefit persisted after adjustment for residual disease (**Figure 4**). Adjuvant therapy was administered in 88.3% (53/60) of patients, predominantly using platinum-based combination regimens.

## 6 Discussion

Ovarian malignancies typically present with nonspecific clinical manifestations, leading to delayed diagnosis, with approximately 70%–90% of cases identified at FIGO stage III or IV. Contemporary multimodal therapeutic strategies—including cytoreductive surgery, platinum-based chemotherapy, and molecularly targeted agents—have shown limited efficacy in advanced-stage disease, with 5-year survival rates remaining below 30%, largely due to frequent extraovarian dissemination at initial diagnosis.

Squamous cell carcinoma (SCC) of the ovary is rare, accounting for fewer than 1% of primary ovarian malignant tumors. Within this spectrum, most cases arise from malignant transformation of pre-existing mature cystic teratomas or, less frequently, from endometriosis or Brenner tumors (1). Metastatic SCC to the ovary—most commonly originating from the cervix, lung, or upper aerodigestive tract—must be rigorously excluded through immunohistochemical profiling (e.g., p16, HPV *in situ* hybridization [ISH])) (2, 3). *De novo* POSCC, arising in the absence of identifiable precursor lesions, represents the rarest subgroup (4, 5).

### 6.1 Etiology and pathogenesis

*De novo* POSCC is exceedingly rare, and its pathogenesis remains poorly understood. Most ovarian SCCs are associated with dermoid cysts, Brenner tumors, or ovarian endometriosis. These tumors are hypothesized to arise from oncogenic stimuli that induce synchronous or metachronous neoplasia in histologically or embryologically related tissues. Yoshida et al. first reported distinct microRNA expression profiles in SCC arising from mature teratomas, identifying two markedly upregulated microRNAs (miR-151a-3p and miR-378a-3p) and two markedly downregulated microRNAs (miR-26a-5p and miR-99a-5p) (6). Subsequently, Yoshida et al. identified unique gene expression signatures in SCC–mature teratoma and proposed KLF5-related factors as potential therapeutic targets (7). However, the molecular characteristics of POSCC remain insufficiently defined because of its rarity. Elucidation of these molecular features may improve understanding of POSCC pathogenesis and guide future therapeutic development.

### 6.2 Preoperative diagnosis

Ovarian neoplasms require systematic evaluation as potentially malignant lesions, irrespective of patient age or tumor size, with histopathological assessment remaining essential for definitive diagnosis. POSCC exhibits a wide age distribution, spanning the third to ninth decades of life (27–90 years). The mean age of 53.4 ± 15.0 years observed in our cohort is consistent with previously reported epidemiological data.

Systematic reviews indicate that 60%–75% of POSCC cases present with nonspecific abdominal or pelvic symptoms, most commonly chronic pelvic pain (58.2%) and palpable adnexal masses (43.7%). In postmenopausal women, the triad of a unilateral pelvic mass, persistent abdominal discomfort exceeding 4 weeks, and elevated CA-125 levels (>35 U/mL) should prompt urgent diagnostic evaluation. These clinical indicators, assessed using transvaginal ultrasonography, CT and/or MRI for locoregional staging, and serum biomarker panels (CA-125, HE4, and ROMA index), frequently correlate with occult malignancy, as supported by multivariate logistic regression analyses referenced in ACOG/SGO guidelines.

### 6.3 Tumor markers

The diagnostic utility of serum biomarkers, including CA-125, CA19-9, carcinoembryonic antigen (CEA), and SCC antigen, in POSCC remains controversial. Although elevated levels (CA-125 >300 U/mL, SCC >2.0 ng/mL) demonstrate sensitivities of 68%–72% for malignancy, specificity is limited because of frequent elevations in benign conditions such as mature teratomas and endometriomas. Multivariate receiver operating characteristic (ROC) analyses indicate poor discriminatory performance for differentiating malignant from benign adnexal masses using conventional cutoff values. Furthermore, serum biomarker levels do not consistently correlate with FIGO stage or histological grade.

High-risk human papillomavirus (HPV) genotypes, particularly HPV16 and HPV18, are established oncogenic drivers in anogenital and oropharyngeal squamous carcinomas through E6/E7-mediated degradation of tumor suppressor proteins p53 and retinoblastoma (Rb). This mechanism results in cell cycle dysregulation, telomerase activation, and resistance to apoptosis—hallmarks that have been detected in approximately 18%–22% of POSCC specimens via PCR or ISH identification of integrated HPV DNA (8–10). Overexpression of p16 (≥70% nuclear and cytoplasmic staining), a surrogate marker of HPV oncogenic activity, shows a 91% concordance with HPV-positive POSCC, compared with 23% in HPV-negative metastatic SCC. These molecular features provide important discriminatory value when evaluating ovarian squamous malignancies of uncertain origin.

### 6.4 Pathological characteristics of POSCC

POSCC is an exceedingly rare malignant epithelial tumor. The histogenesis of ovarian SCC remains incompletely elucidated, with more than 80% of cases arising from malignant transformation of ovarian mature teratomas. Rare cases have also been reported in association with endometriosis or Brenner tumors. POSCC is characterized by high malignancy and a generally poor prognosis. Definitive diagnosis requires exclusion of metastatic squamous cell carcinoma from other primary sites, particularly the cervix.

Histologically, POSCC is typically composed of large polygonal squamous cells with eosinophilic cytoplasm and intercellular bridges, with or without keratin pearl formation. Hallmark proliferative features include high cellularity, frequent mitotic activity, and marked pleomorphism. In tumors associated with dermoid cysts, the presence of derivatives from all three germ layers surrounding the squamous cell carcinoma (SCC) component supports malignant transformation of a teratoma. In cases arising from endometriosis, iron staining may demonstrate hemorrhage and hemosiderin deposition, providing supportive diagnostic evidence.

Immunohistochemical staining (IHC) plays a critical role in confirming primary ovarian origin and excluding other tumors. (i) Core POSCC markers: Positive staining for high-molecular-weight cytokeratins (e.g., 34βE12, CK5/6) and p63 is characteristic. (ii) Markers for differential diagnosis: p16, WT1, and p53 assist in distinguishing POSCC from other gynecologic malignancies and from squamous metaplasia. Approximately 80% of POSCC cases exhibit mutant-type p53 expression, while membranous WT1 positivity has been reported in a subset of tumors. Strong p16 expression suggests a cervical primary, as cervical squamous cell carcinoma typically shows diffuse p16 positivity with wild-type p53 expression.

### 6.5 Surgery and adjuvant treatment

Early-stage detection of POSCC allows for R0 cytoreduction and timely initiation of multimodal therapy, thereby significantly improving 5-year survival rates. As demonstrated in the present study, the surgical management of POSCC follows principles established for epithelial ovarian cancer. Complete cytoreduction is consistently associated with improved prognosis. In particular, patients undergoing radical hysterectomy with bilateral salpingo-oophorectomy (BSO) combined with systematic lymphadenectomy achieved superior progression-free survival compared with those treated with BSO alone.

Selection of optimal adjuvant therapy to complement surgical treatment is essential for improving patient outcomes. However, postoperative radiotherapy has not been shown to confer a long-term survival benefit (11). Because of the rarity of POSCC, no international consensus exists regarding the efficacy of adjuvant chemotherapy after surgery. As POSCC is frequently treated analogously to epithelial ovarian cancer, platinum-based chemotherapy combined with paclitaxel is commonly recommended (3, 4, 12). Nevertheless, the therapeutic benefit of this regimen remains controversial, with conflicting evidence reported in the literature (13–18).

Therapeutic insights derived from the management of SCC in other organs may inform future strategies for POSCC. Vascular endothelial growth factor (VEGF) is a key mediator of tumor angiogenesis, and agents such as bevacizumab have demonstrated antitumor efficacy in several SCCs, including head and neck and cervical cancers. In addition, the advent of immunotherapy offers novel treatment opportunities. Consequently, investigation of targeted therapies and immunotherapeutic approaches as components of first-line treatment for newly diagnosed POSCC appears both promising and urgently warranted.

### 6.6 Stage and prognosis

Tumor stage and histological grade at initial diagnosis serve as independent prognostic indicators in POSCC (19). Five year overall survival (OS) rates decline markedly across FIGO stages. The reported 5-year survival rate for stage I disease is 76%, whereas corresponding rates for advanced stages are 34%, 21%, and 0% for stages II, III, and IV, respectively (4, 13). The aggressive biological behavior of POSCC is reflected by frequent transserosal spread, with most cases demonstrating parametrial, rectosigmoid, or pelvic peritoneal involvement at diagnosis. These invasive patterns necessitate radical cytoreductive surgery as the cornerstone of treatment, typically including radical hysterectomy and bilateral salpingo-oophorectomy, with or without lymphadenectomy and omentectomy. Multicenter analyses have identified optimal debulking (residual disease <1 cm) as the strongest predictor of disease-free survival in advanced POSCC (FIGO stages III and IV), surpassing the prognostic impact of adjuvant treatment modalities. Additional independent adverse prognostic factors include advanced age (≥60 years), large tumor diameter (≥15 cm), and lymphovascular space invasion (4, 12, 20).

## 7 Limitations

This retrospective analysis has inherent methodological limitations that warrant cautious interpretation. First, potential underascertainment of published POSCC cases may introduce selection bias, particularly for rare histological subtypes. Second, incomplete documentation of key prognostic variables, including tumor differentiation grade and maximum tumor diameter, limits the robustness of multivariable modeling. Additionally, the extended temporal span of the included studies (1964–2022) encompasses substantial changes in diagnostic criteria, surgical techniques, and adjuvant therapy protocols. These chronological variations complicate direct outcome comparisons and highlight the need for future prospective registries using standardized data collection frameworks.

## 8 Conclusions

POSCC represents a distinct clinicopathological entity that requires specialized diagnostic and therapeutic strategies. Early-stage detection through advanced imaging, biomarker assessment, and molecular profiling is critical for improving survival outcomes, with stage I disease demonstrating 5-year survival rates exceeding 70%, compared with less than 25% in advanced stages.

The current evidence base is limited by (i) data scarcity, as few reported cases include comprehensive biomarker or genomic profiling; (ii) therapeutic uncertainty, owing to the absence of randomized controlled trials comparing treatment modalities in POSCC; and (iii) staging limitations, as existing FIGO criteria do not incorporate histology-specific prognostic stratification. To address these challenges, we support the Global Consortium for Gynecologic Oncology Innovation (GCGI) roadmap, which emphasizes (i) establishment of multinational POSCC registries with standardized molecular data capture, (ii) development of disease-specific staging frameworks incorporating circulating tumor DNA monitoring, and (iii) prioritization of basket trials evaluating angiogenesis inhibitors and immune checkpoint blockade in SCC histology. Coordinated efforts in these areas may facilitate precision oncology approaches tailored to the unique biology of POSCC and help bridge the current evidence–practice gap.

## Data Availability

The original contributions presented in the study are included in the article/**Supplementary Material**. Further inquiries can be directed to the corresponding author.

## Supplementary material

Supplementary Figure 1 The age distribution of PSCC for 75 cases.

Supplementary Figure 2 Association between patient’s age and tumor size.

Supplementary Figure 3 Literature screening flow chart.
