# Supplementary figures and images for: Case Report: Diagnosis and management of primary ovarian squamous-cell carcinoma: a report of two cases and systematic review of the literature
Source: Front Oncol. 2026 Jan 23;16:1706736. doi: 10.3389/fonc.2026.1706736 (PMC12875973; doi:10.3389/fonc.2026.1706736)

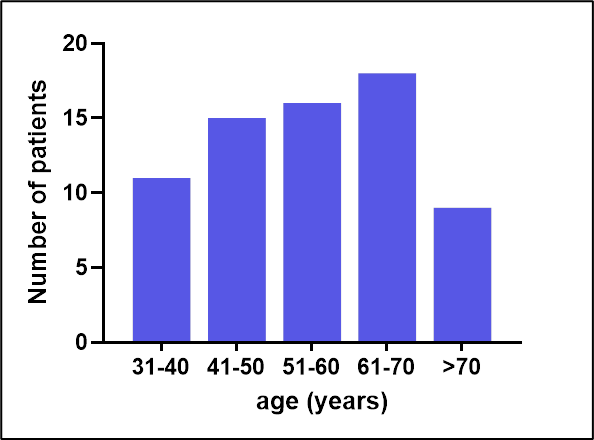

Supplement: Supplementary Figure 1 — The age distribution of PSCC for 75 cases. [file Image1.tif]

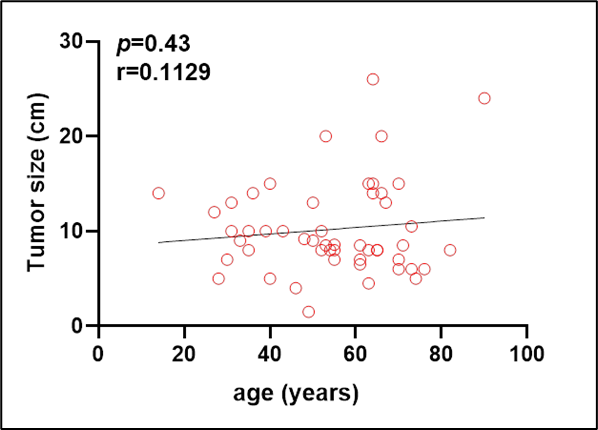

Supplement: Supplementary Figure 2 — Association between patient’s age and tumor size. [file Image2.tif]

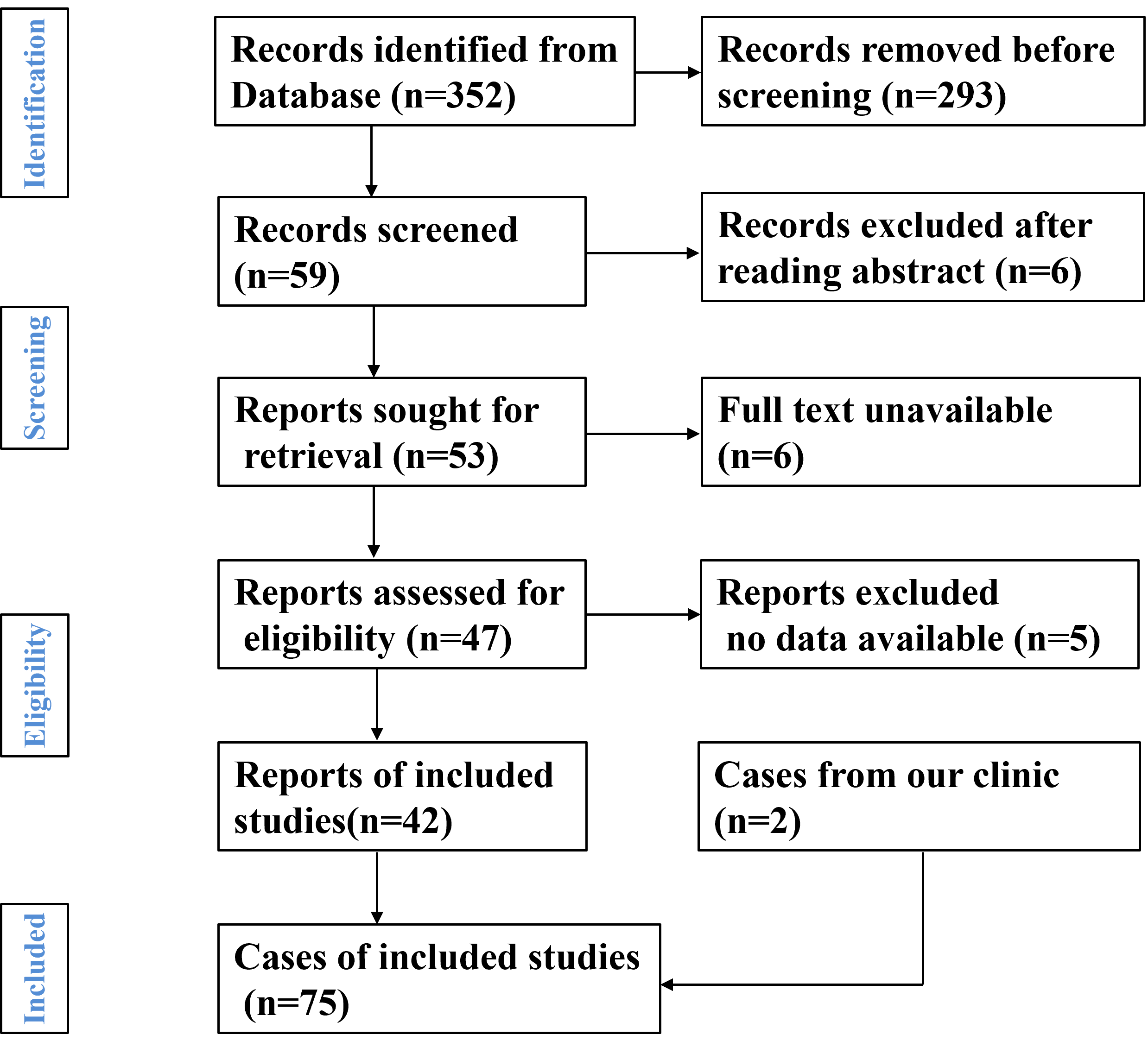

Supplement: Supplementary Figure 3 — Literature screening flow chart. [file Image3.png]
