# Supplementary material for: Case Report: Diagnosis and management of primary ovarian squamous-cell carcinoma: a report of two cases and systematic review of the literature
Source: Front Oncol. 2026 Jan 23;16:1706736. doi: 10.3389/fonc.2026.1706736 (PMC12875973; doi:10.3389/fonc.2026.1706736)
Supplement: Supplementary file 4 [file Supplementaryfile1.docx]

**Suppl. Table 1.** **The age distribution of PSCC for 75 cases.**

| **Age (years)** | **⩽20** | **21-** | **31-** | **41-** | **51-** | **61-** | **>70** | **Total** |
| --- | --- | --- | --- | --- | --- | --- | --- | --- |
| **NO. of Cases** | 1 | 4 | 11 | 15 | 16 | 19 | 9 | 75 |
| **Percentage** | 1.33% | 5.33% | 14.67% | 20.00% | 21.33% | 25.33% | 12.00% | 100% |
